# Application of the Akaike Information Criterion to Ultrasonic Measurement of Liquid Volume in a Cylindrical Tank

**DOI:** 10.3390/s25237191

**Published:** 2025-11-25

**Authors:** Krzysztof J. Opieliński, Tomasz Świetlik

**Affiliations:** Department of Acoustics, Multimedia and Signal Processing, Faculty of Electronics, Photonics and Microsystems, Wroclaw University of Science and Technology, Wybrzeże Stanisława Wyspiańskiego 27, 50-370 Wrocław, Poland; tomasz.swietlik@pwr.edu.pl

**Keywords:** ultrasonic measurement, liquid volume, cylindrical tank, Akaike Information Criterion (AIC), amplitude threshold method

## Abstract

The ultrasonic sensor method is the most significant and widely accepted technique for measuring liquid levels in tanks. Ultrasonic waves are particularly advantageous in the case of explosive, flammable, or aggressive liquids because of the possibility of introducing ultrasonic pulses through the tank wall safely. Often, the measurement of these liquids should be performed automatically using electronic devices to ensure that the tank remains sealed. In the case of ultrasound, measurements are made using the echo method, with a transmitting-receiving (transceiver) ultrasonic transducer that sends vibration pulses into the tank. The measured delay between the transmitted pulse and the pulse reflected from the liquid surface is proportional to the liquid level in the tank. The volume of liquid can be calculated on the basis of the dimensions of the tank. In this method, it is very important to accurately determine the delay by detecting the beginning of the reflected pulse, which determines the accuracy of the measurement of the level of the liquid and its quantity in the tank. To improve this accuracy, this paper proposes the use of the Akaike Information Criterion (AIC) used in statistics for model selection. As part of the research, ultrasonic test measurements were performed for a tank filled with water and extraction gasoline. This allowed a favorable comparison of the AIC method with the most commonly used threshold method and for determining the accuracy of liquid volume measurements in the tank using both methods in relation to the parameters of several selected ultrasonic sensors. The accuracy obtained using the AIC method was found to be better than that of the fixed-fractional amplitude threshold method. Furthermore, the AIC method is more versatile because it is less sensitive to interference and is capable of detecting the onset of a pulse regardless of its shape and frequency, even in noise. It is suitable for real-time embedded systems for liquid level measurement as well.

## 1. Introduction

Monitoring liquid levels and controlling liquid volumes in tanks is crucial wherever liquids need to be stored and processed in a variety of industries. Liquid level monitoring methods have a wide range of applications, from wastewater treatment plants and the petrochemical industry to food processing plants, from pharmaceutical laboratories and biochemical processing to industrial silos [1,2]. Precise measurements not only ensure process efficiency and safety, but also contribute to cost optimization and prevent raw material waste. In this context, the development of measurement technologies plays a key role in enabling precise monitoring and control of liquid levels and quantities in tanks. Based on a review of the literature, it can be observed that most of the research methods on this topic were developed before 2010, but between 2012 and 2017, 44 articles were published, and between 2018 and 2022, up to 74 articles were published in various scientific journals [3]. This shows that research on liquid-level methods and sensors has increased dramatically over the past five years and is currently a desirable area of research.

Various electronic sensors are used to measure the volume of liquids in tanks by measuring their level of fill. These sensors operate using various physical phenomena, such as magnetostriction, the generation of ultrasonic waves, microwaves and light, changes in electrical capacitance, and changes in hydrostatic pressure. The most commonly used are hydrostatic probes, capacitive and floating level sensors, pressure transducers, optical sensors, microwave radar gauges, and ultrasonic transducers [4]. These can be sensors mounted inside or outside the tank, immersed or not immersed in the liquid, and floating on its surface. Liquid level sensors are classified in various ways [3]. Based on the relationship between the user (operator) and the measurement, level sensors can be classified as contact and non-contact measurements. The sensor can also perform continuous or point measurements. Point-level measurement indicates a measurement taken as high or low, empty, or available in relation to switches/circuit breakers and protective alarms, e.g., to prevent leaks. Furthermore, the point measurement does not provide any information on the amount of liquid in the tank. Continuous liquid level measurement, on the other hand, allows the volume to be monitored over time. Level sensors can also be classified as direct—measuring directly, and indirect—measuring liquid parameters and estimating its volume based on these parameters. Various classifications of the most commonly used liquid level sensors are shown in [3]. The same source also provides a description of how they work.

Liquid level sensors have parameters that vary greatly depending on the manufacturer, mode of operation and intended applications, providing liquid level measurement accuracy 0.1–1.0 mm, measurement resolution 0.012–0.1 mm, and measurement repeatability 0.05–0.3 mm. Internal magnetostrictive floating probes are most commonly used in gas station fuel tanks due to their ability to monitor fuel level simultaneously and continuously, as well as the parameters and vapors remaining in the tank [5]. This is also particularly justified due to the need for access to the probe, which must be calibrated or replaced if necessary.

The most significant measurement technique accepted by various researchers is the ultrasonic level sensor [6], which uses ultrasonic waves to measure liquid levels. The use of ultrasound is very advantageous when measuring the level of liquids, e.g., explosive, flammable, or aggressive, due to the possibility of safely introducing these waves through the wall of the tank from the liquid side, i.e., from the bottom of the tank. In this method, the level and volume of liquid in the tank are measured using a sensor in the form of a piezoelectric transducer. The sensor sends a short sinusoidal pulse of a specific frequency. The pulse reflects off the surface of the liquid and returns to the sensor. The essence of measuring the amount of liquid in the tank is to precisely measure the time between the start of the transmitted and received ultrasonic wave pulses (echoes). The time it takes for the ultrasonic signal to travel from the transmitter to the receiver is defined as the transit time or the time of flight (TOF). Due to energy loss, time shift, and frequency dispersion during propagation, the actual received ultrasonic pulse will always exhibit waveform changes and noise interference, making it challenging to achieve satisfactory accuracy using traditional methods. In addition, many factors influence the accuracy and errors of the measurement, including the operating frequency and size of the sensor, which determine the length and shape of the pulse. Therefore, the accurate determination of ultrasonic TOF is a key technique in ultrasonic applications [7], particularly in measuring the level of fill of liquid tanks. For example, for a large horizontal cylindrical fuel tank with a capacity of 120 m^3^ (radius *R* = 1.5 m, height or length *H* = 16.98 m) [8], when filled halfway and with a liquid level measurement error in the tank of only Δ*h_m_* = ±1 mm, the liquid volume measurement error can be estimated as Δ*V_l_* ≈ ±51 dm^3^. For the same vertically positioned tank, the error in measuring the volume of liquid does not depend on the filling of the tank and is Δ*V_l_* ≈ ±7 dm^3^.

The basic ultrasonic method used to measure liquid levels in tanks is the threshold detection method, which is also used in radar systems and identifies the received signal when the echo amplitude exceeds a set threshold level [7,9] or reaches its maximum value (peak method). Many improved threshold methods have also been developed, such as the double threshold method [10], the dynamic threshold method [11], and the variable coefficient threshold method [12]. Although threshold methods are the simplest, they are also the most susceptible to interference caused by noise and changes in the shape of the detected pulse.

Another category of TOF measurement methods includes correlation methods [13], which were first used in radar technology. Later, more advanced correlation methods were proposed and applied in TOF measurements, such as frequency modulation-based cross-correlation [14], phase correlation method [15], and cross-correlation with sine wave matching techniques [16]. Although correlation methods are less susceptible to interference, their accuracy is limited by the sampling frequency and requires real-time reference signal updates according to different application conditions, causing deviations in TOF detection.

The third category includes the most accurate but also the most complex methods of TOF estimation based on parameter estimation using an accurate ultrasonic echo model [17,18] and efficient and accurate optimization algorithms [19,20,21]. In these methods, a reasonable empirical model of the ultrasonic signal is first selected, and then efficient algorithms are used to fit the received ultrasonic signal to obtain the transit time. These methods ensure not only high measurement accuracy, but also strong resistance to interference [22]. Nevertheless, they require extensive calculations and costly digital signal processing, which significantly degrades their real-time performance. Furthermore, the convergence results of these methods are highly dependent on good initialization, and the echo model used for fitting must be updated under different application conditions, which negatively affects real-time performance.

The methods of estimating the energy distribution in the ultrasonic signal can be used to calculate the TOF of ultrasonic pulses, instead of threshold methods. Such methods allow for the separation of different types of waves from the acoustic signal too and are used in geophysics to detect seismic waves generated by earthquakes. These signals contain P-waves and S-waves. P-waves are faster compression waves that arrive first, whereas S-waves are slower shear waves that arrive later and are often more destructive. A review, testing, and evaluation of the precision of such methods as applied to arrival time detection for downhole microseismic data can be found in [23]. These methods can be classified as single-level window-based methods, which require the window size and location to be specified in order to compute the data, e.g., energy ratio (ER) methods, single-level non-window-based methods (e.g., Akaike information criterion), multi-level methods (from multiple sensors or multiple locations) or matrix-based methods (e.g., cross-correlation-based approaches) and hybrid methods combining several single-level methods (e.g., Akazawa method) [23].

There are also many hybrid algorithms in the literature that combine information from different individual algorithms to obtain more accurate and precise TOF measurements [24,25,26,27,28,29,30,31,32,33].

A review of the literature on many different measurement methods confirms the view that no single algorithm is optimal for all conditions, and the accurate determination of TOF remains a challenge. It depends on the specific application (device, implementation, computational complexity, measurement signal parameters). Therefore, understanding the parameters and limitations of these algorithms can help improve data processing results. Similarly, knowing the speed of any algorithm is important, especially if the goal is to perform real-time data analysis to provide up-to-date feedback during measurements. Based on the review of various algorithms above, it was found that the information criteria used in statistics for model selection can be used to precisely determine the pulse transit time in this method. The most popular information criteria are the Akaike Information Criterion (AIC) and the Bayesian Schwartz Criterion (BIC) [34]. These criteria are considered the most reliable tests of the type and structure of the model and are simple to calculate. They can be adapted for automatic detection of the start of the receiving pulse, which may allow independence from the pulse parameters. The AIC algorithm has been tested to detect the onset of pulses in in vivo breast imaging using ultrasound transmission tomography (UTT) [35,36]. However, TOF measurements using the Akaike method proved to be insufficiently accurate due to the phenomenon of ultrasound beam refraction in this application [37]. This phenomenon causes small-amplitude disturbances before the actual start of the pulse. However, such interference does not occur when measuring the level of a quasi-homogeneous liquid in a tank. The liquid has a large surface area, the reflection coefficient at the boundary is high, and the attenuation of ultrasound in the liquid is low; therefore, the pulse energy and the signal-to-noise ratio (SNR) are high. The rise time of the pulse reflected from the liquid surface is short and undisturbed. In addition, unlike seismic signals, for example, the signal reflected from the liquid surface is uncomplicated and consists only of single, short pulses reflected multiple times from the liquid surface. The detection of the first echo is easy in this case.

Therefore, it can be assumed that the AIC method can be useful for measuring liquid levels in embedded systems operating in real time and that it can be more accurate and versatile in various measurement conditions than threshold methods, which was the aim of this research.

In this paper, the authors tested several ultrasonic piezoelectric transducers with different operating frequencies, which generate different pulses in measurements of the liquid level in a cylindrical tank. It is usually important to choose the right ultrasonic sensor and operating frequency for accurate measurement. It is known that too low a frequency when measuring time based on a fixed pulse amplitude threshold causes large errors. On the other hand, too high a frequency worsens pulse detection, which is more attenuated and reduces SNR. It will be interesting to see whether automatic measurement using the AIC method, which is little known in such applications, will enable accurate liquid level measurements to be taken in a more universal way. In this paper, ultrasonic test measurements were performed for a cylindrical tank filled with water and extraction gasoline. A horizontally placed tank was selected because of the more difficult method of measuring and converting the liquid level in the tank to its occupied volume. This allowed for a comparison of the AIC method with the most commonly used threshold method and for an estimation of the accuracy of level and liquid volume measurements in the tank using these methods in relation to the parameters of several selected ultrasonic sensors. This is a novelty compared to other studies in the field of TOF detection. The results of the measurements and calculations showed that the AIC method is less sensitive to interference and changes in the shape of ultrasonic pulses reflected from the surface of the liquid and allows for automatic determination of the volume of liquid in the tank with better accuracy than the threshold method. It has also been demonstrated that the computational complexity of the AIC algorithm is low and is suitable for real-time embedded systems for liquid level measurement.

## 2. Materials and Methods

### 2.1. Method and Conditions of Measurements

**Figure 1:**
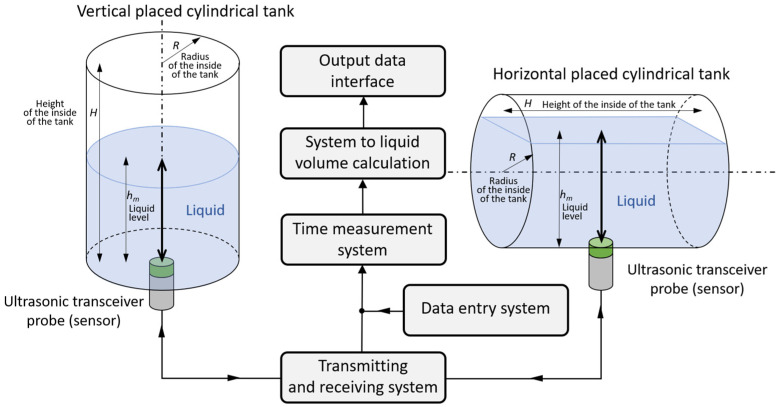
Method of measuring the volume of liquid in a cylindrical tank for two cases of tank positioning: vertical and horizontal.

Figure 1 shows how to measure the volume of liquid in a cylindrical tank for two cases of tank positioning, vertically and horizontally, respectively.

**Figure 2:**
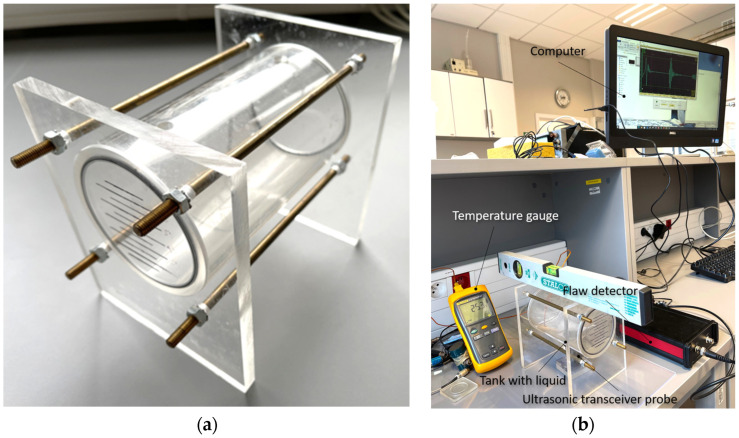
View: (**a**) of a horizontally positioned tank and (**b**) of the set-up used to measure the quantity of liquid in this tank.

This paper is limited to the more difficult method of measuring the amount of liquid in a horizontally positioned tank (Figure 2). The tank is made of polymethyl methacrylate (organic glass) with a flat undercut at the bottom, allowing mechanical coupling across the entire surface of the ultrasonic sensor with the tank wall using a thin layer of oil, gel, or grease. The thickness of the tank wall between the surface of the ultrasonic sensor and the liquid in the tank is 2 mm. The radius of the tank interior (cylinder radius) is *R* = 40 mm, and the height of the tank interior (cylinder height) is *H* = 142 mm. The propagation speed of the ultrasonic wave in the tank material is 2670 m/s.

Measurements were performed using a device consisting of a selected ultrasonic transceiver probe and four main blocks: a transmitting and receiving system, a time measurement system, a data entry system, and a system for calculating the volume of liquid in the tank (Figure 1). This role was carried out by a prototype mobile single-channel ultrasonic flaw detector with an output data interface enabling recording of received ultrasonic pulses reflected from the liquid surface in the form of digital signals. The transceiver probe was placed under the bottom of the tank, in the geometric center of this surface (Figure 2b). Due to this, the tank did not have to be very precisely leveled. The transmitting and receiving system generates a short voltage pulse that is sent by the transceiver probe in the form of an ultrasonic wave into the tank with liquid from the bottom. The ultrasonic pulse passes through the tank wall and the liquid, then reflects off the surface of the liquid in the form of an echo, and returns to the transceiver probe after passing through the tank wall again. The time measurement system measures the delay between transmitted and reflected pulses, which is proportional to the distance between the probe and the surface of the liquid in the tank. The data entry system is used to enter the values necessary to calculate the volume of liquid in the tank: tank dimensions, correction for tank wall thickness, temperature correction, and ultrasonic wave speed values. The volume of liquid in the tank is calculated on the basis of the time measurement and the data entered.

During the measurements, the temperature of the liquid in the tank was controlled using a thermocouple with an accuracy of ±0.1 °C (Figure 2b). The temperature of distilled water during the measurement of different amounts of it in the tank varied within a maximum range of 24.8–25.7 °C. The propagation speed of the ultrasonic wave in distilled water to convert the measured transit time values into distance was determined for each measurement of the level in the tank depending on the temperature, using the empirical formula proposed by Marczak [38], expressed as a fifth degree polynomial, whose coefficients were fitted by the least squares method to 209 measurement values obtained by Del Grosso and Madera [39], Kroebel and Mahrt [40] and Fujii and Masui [41]:

(1) $$c_{w}\;=\;\sum_{i=0}^{5}k_{i}{\;\cdot \;T}^{i},$$

where *T* is the temperature in the range of 0–100 °C, *k_i_* are coefficients listed in Table 1.

Formula (1) allows the propagation speed of an ultrasonic wave in distilled water to be determined with an uncertainty of several dm/s. The temperature of the extraction gasoline during the measurement of different amounts in the tank varied within a narrow range of 25.1–25.6 °C. In this case, a constant value of ultrasound propagation speed was assumed in this liquid, *c_eg_* ≈ 1120 m/s, based on ultrasonic transmission measurements at a temperature of 25 °C.

### 2.2. Ultrasonic Sensors

**Figure 3:**
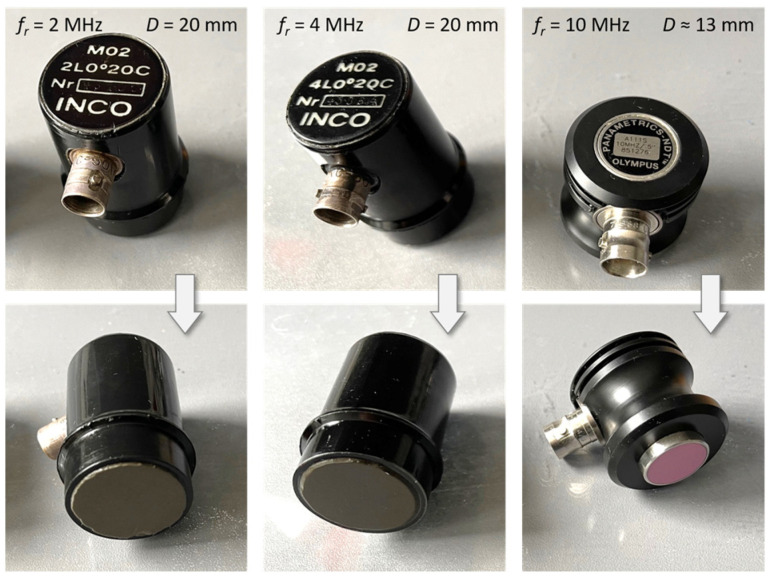
Ultrasonic sensors used for measurements.

Three different ultrasonic sensors were used for the measurements in the form of transmitting-receiving (transceiving) piezoelectric probes with piezoceramic plates (C) vibrating in thickness mode (Figure 3), designed for non-destructive ultrasonic testing and generating a longitudinal wave (L) penetrating the medium perpendicular to the active surface of the probe (angle of incidence 0°): INCO2L0°20C (resonance frequency *f_r_* = 2 MHz, active surface diameter *D* = 20 mm), IN-CO4L0°20C (resonance frequency *f_r_* = 4 MHz, active surface diameter *D* = 20 mm), PANAMETRICS-NDT A111S10MHZ/.5” (resonance frequency *f_r_* = 10 MHz, active surface diameter *D* ≈ 13 mm).

The ultrasonic transducers were powered by spike pulses with an amplitude of 100 V and a duration of 80 μs from a prototype ultrasonic flaw detector with an output data interface. The flaw detector allowed reception echoes to be amplified to 80 dB. The signal sampling frequency was 100 MHz, and its amplitude was quantized with a resolution of 8 bits (256 levels). To eliminate noise and interference, the appropriate bandpass filters were connected to the flaw detector receiving channel for the individual transducers: 0.5–5 MHz and 5–20 MHz. In the recorded signals reflected from the liquid surface, the SNR was not less than 55 dB. High SNR is typical in liquid level measurements.

### 2.3. Calibration and Algorithm for Calculating the Volume of Liquid in a Tank

**Figure 4:**
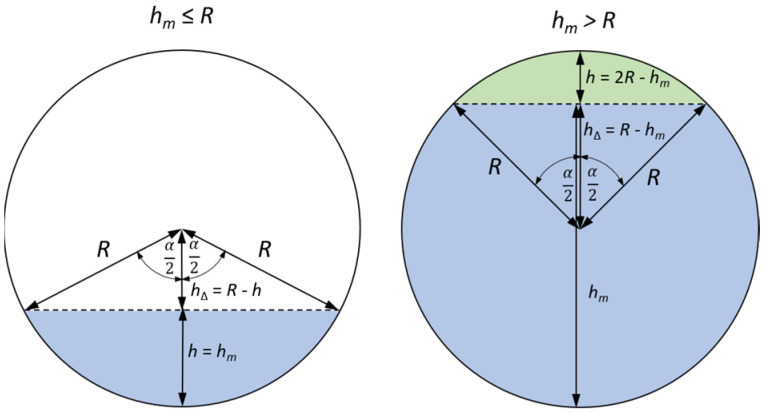
Geometry illustrating how to develop an algorithm for determining the volume of liquid in a horizontally positioned tank based on determining the surface area of a circle segment.

Individual levels of liquids tested *h_m_* in the tank were determined for measurement purposes, according to the markings on the side wall of the tank (Figure 2), by dispensing reference volumes of liquid *V_REF_* using laboratory vessels with an accuracy of 5 mL (Δ*V_REF_* = ±2.5 mL). Based on the measured times of returning ultrasonic pulses reflected from the liquid surface, the actual measured liquid levels *h_m_* were calculated using a formula that takes into account the passage of the ultrasonic wave through the tank wall:

(2) $$h_{m}\;=\;\left(\frac{1}{2}\cdot t_{m}-\frac{W_{t}}{c_{t}}\right)\cdot c_{l},$$

where *t_m_*—time to return of the ultrasonic pulse reflected from the surface of the liquid, *W_t_*—thickness of the tank wall between the surface of the ultrasonic sensor and the liquid in the tank, *c_t_*—speed of propagation of the ultrasonic wave in the tank material, *c_l_*—speed of propagation of the ultrasonic wave in the tested liquid. To determine the volume of liquid in the tank *V_l_* based on the liquid levels *h_m_* determined from the measurements of the echo return time *t_m_*, an algorithm was developed to calculate the surface area of a circle segment for two possible cases: when the liquid level is less than or equal to the inner radius of the cylindrical tank (*h_m_* ≤ *R*) or greater than it (*h_m_* > *R*) (see Figure 4).

For the case *h_m_* ≤ *R*, the area of the circle segment (*S_CSegment_*) occupied by the liquid in a horizontally positioned tank can be determined as the difference between the area of the circle slice (*S_CSlice_*) and the area of the isosceles triangle (*S_ITriangle_*) with sides *R*, using the formula (Figure 4):

(3) $$S_{CSegment}\;=\;S_{CSlice}-S_{ITriangle}=\frac{1}{2}R^{2}\left(\alpha -sin\alpha \right)\;,$$

where *R* is the inner radius of the side wall of the tank with a circular cross-section, and *α* is the angle of the circle slice.

The angle of the circle slice (sector) *α* can be calculated using the formula:

(4) $$\alpha \;=\;2\;arccos\left(\frac{h_{∆}}{R}\right)\;,$$

where *h*_Δ_ = *R* − *h_m_* is the height of the isosceles triangle that defines the circle slice. Based on formulas (3) and (4), the volume of liquid in the tank for the case *h_m_* ≤ *R* can be calculated as:

(5) $$V_{l}\;=\;{H\cdot S}_{CSegment}=R^{2}\cdot \left(arccos\left(\frac{R-h_{m}}{R}\right)-\frac{1}{2}\sin\left(2\;arccos\left(\frac{R-h_{m}}{R}\right)\right)\right)\;.$$

For the case where *h_m_* > *R*, the area of the *S_CSegment_* occupied by the liquid in a horizontally positioned tank can be determined as the difference between the area of the circle and the area of the circle segment not occupied by the liquid in the tank (the circle segment in green in Figure 4):

(6) $$V_{l}\;=\;H\cdot \left({\pi R^{2}-S}_{CSegment}\right)=H{\cdot R}^{2}\left(\pi -arccos\left(\frac{R-h_{m}}{R}\right)+\frac{1}{2}\sin\left(2\;arccos\left(\frac{R-h_{m}}{R}\right)\right)\right)\;.\;$$

### 2.4. Echo Return Time Detection Using the Threshold Method

**Figure 5:**
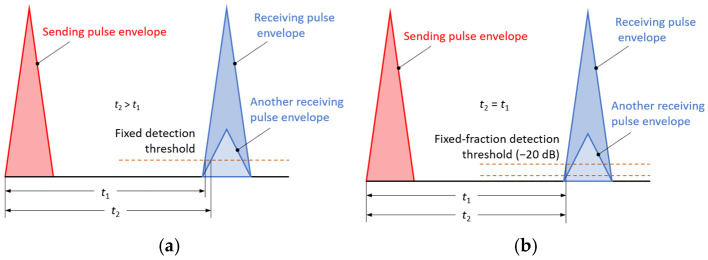
Illustration of the impact of detection type on transit time measurement error: (**a**) fixed signal detection threshold, (**b**) fixed-fractional signal detection threshold.

In transmission and reflection measurements of transit time (sender → detector) and return of ultrasonic pulses (sender → reflector → receiver), threshold methods are the most widely used [42,43]. Received pulse detection can be performed with a fixed or so-called fixed-fractional (percentage) amplitude threshold, as shown in Figure 5. When measuring the transit or return time of a pulse, the fragment of the received signal limited by the measurement gate is searched until a sample exceeding the set threshold is encountered. Ultrasonic signal pulses have a certain non-zero rise time (depending, among other things, on the operating frequency of the ultrasonic transducer) [44]. A fixed detection threshold may be a source of errors resulting from changes in the amplitude of the received signal caused by attenuation in the tested medium; the results of the transit time measurements will then depend on the threshold set (Figure 5a). Therefore, a fixed-fractional variant has been introduced in the algorithm developed to measure the return time of a pulse reflected from the liquid surface. Detection of the received signal with a fixed-fractional (percentage) threshold involves measuring the maximum amplitude of the received signal, setting the detection threshold in relation to this amplitude (above the noise), and measuring the pulse transit time. The detection threshold in fixed-fraction time measurement is therefore automatically selected as a certain fraction of the maximum amplitude of the received pulse, above noise and interference. For example, for a fraction of 0.1 (that is, −20 dB amplitude level), the threshold will always be 10% of the pulse amplitude and, if this amplitude changes, the absolute detection threshold will also change (Figure 5b). In the fixed-fractional method, the detection threshold does not depend on the signal amplitude at the detection point, which eliminates transition time measurement errors caused by its changes.

However, the fixed-fractional method of measuring the transit or return time of a pulse introduces time estimation errors that increase with the level of noise, because the transit time is determined at the point where the signal amplitude exceeds the noise amplitude. The actual start of the received signal is hidden in the noise, so the values measured in this way are overestimated depending on the SNR.

### 2.5. Echo Return Time Detection Using the AIC Method

The popular Akaike Information Criterion (AIC) used in statistics for model selection [44] can be used for automatic detection of pulse transit or return times in ultrasonic transmission and reflection measurement methods.

In this paper, the algorithm developed according to the AIC [45] was used for the automatic detection of the return time of ultrasonic pulses reflected from the surface of the liquid in the tank. A commonly used method to obtain the AIC function is the VAR-AIC method proposed by Maeda [46]. The AIC picker is a widely used method to pick the onset time of the impact signal, which has been widely applied in the detection of the arrival time of seismic waves [47]. This algorithm was adapted to the measurement requirements. The measurement window in which the signal was recorded contains *N* samples, which can be marked with indices *i* = 1, 2, …, *N*. This window is then divided into two sections (sub-windows) with a variable number of samples *k* = 2, 3, …, *N* − 1, in such a way that section No. 1 marked as *S*(1, *k*) contains the initial samples *i* = 1, 2, …, *k* from the main window, while section No. 2 marked as *S*(*k* + 1, *N*) contains the remaining samples *i* = *k* + 1, 2, …, *N* from the main window. The *AIC*(*k*) values for *k* = 2, 3, …, *N* − 1 are then calculated using the formula:

(7) $$AIC\left(k\right)=k\cdot log\left[var\left(S\left(1,k\right)\right)\right]+\left(N-k-1\right)\cdot log\left[var\left(S\left(k+1,N\right)\right)\right]\;,$$

where $var\left(S\left(1,k\right)\right)$—variance of sample values in sub-window $S\left(1,k\right)$, $var\left(S\left(k+1,N\right)\right)$—variance of sample values in sub-window $S\left(k+1,N\right)$ in the form:

(8) $$var\left(S\left(1,k\right)\right)=\sigma_{k-1}^{2}=\frac{1}{k-1}\sum_{l=1}^{k}{\left(S\left(l,l\right)-\bar{S}\right)}^{2}\;,$$

(9) $$var\left(S\left(k+1,N\right)\right)=\sigma_{N-k-1}^{2}=\frac{1}{N-k-1}\sum_{l=k+1}^{N}{\left(S\left(l,l\right)-\bar{S}\right)}^{2}\;,$$

where $\bar{S}$ denotes the mean value of the samples. In the *S*(1, *k*) window, the mean value $\bar{S}$ is calculated using the formula:

(10) $$\bar{S}=\sum_{i=1}^{k}\frac{samples\left(i\right)}{k}\;,$$

and in window $S\left(k+1,N\right)$ the mean value $\bar{S}$ is calculated using the formula:

(11) $$\bar{S}=\sum_{i=k+1}^{N}\frac{samples\left(i\right)}{N-k}\;.$$

The return time of the ultrasonic pulse reflected from the surface of the liquid in the tank is determined for the sample from the window for which the *AIC*(*k*) value reaches its minimum.

## 3. Measurements and Calculations

**Figure 6:**
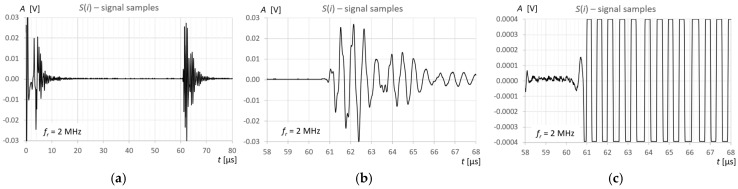
Example recording of ultrasonic pulses reflected from the surface of water in a horizontally placed tank (approximately 45 mm from the bottom, *V_REF_* = 410 mL) for the 2 MHz transducer, sequentially in (**a**) long window, (**b**) measurement window, and (**c**) zoomed window.

**Figure 7:**
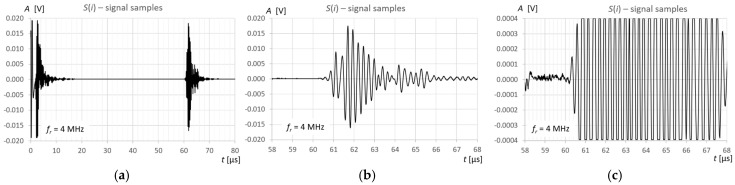
Example recording of ultrasonic pulses reflected from the surface of water in a horizontally placed tank (approximately 45 mm from the bottom, *V_REF_* = 410 mL) for the 4 MHz transducer, sequentially in (**a**) long window, (**b**) measurement window, and (**c**) zoomed window.

**Figure 8:**
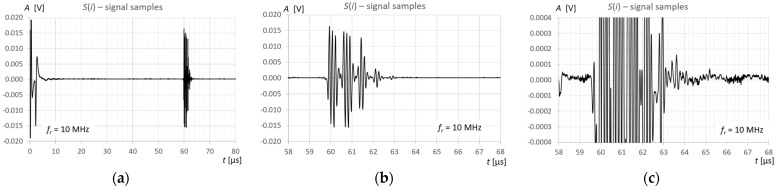
Example recording of ultrasonic pulses reflected from the surface of water in a horizontally placed tank (approximately 45 mm from the bottom, *V_REF_* = 410 mL) for the 10 MHz transducer, sequentially in (**a**) long window, (**b**) measurement window, and (**c**) zoomed window.

**Figure 9:**
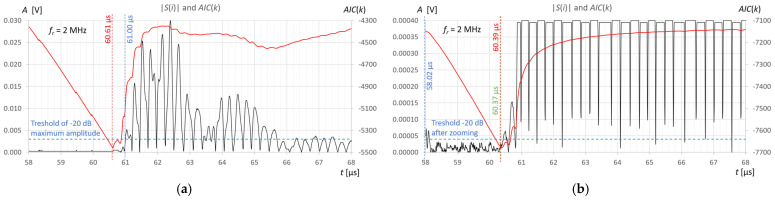
Example results of detecting the start of a pulse reflected from the surface of water in a horizontally placed tank (approximately 45 mm from the bottom, *V_REF_* = 410 mL) for 2 MHz ultrasonic transducer using the fixed-fractional threshold method (blue), AIC method (red) and subjective method of searching for the first zero-crossing (green), in the measurement window (**a**) and zoomed window (**b**).

**Figure 10:**
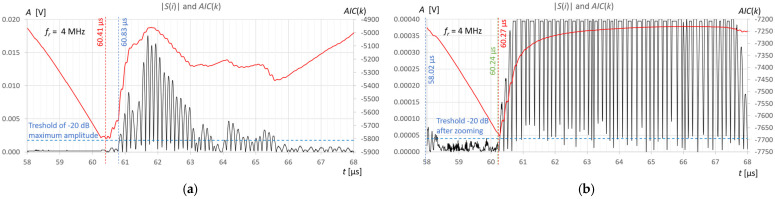
Example results of detecting the start of a pulse reflected from the surface of water in a horizontally placed tank (approximately 45 mm from the bottom, *V_REF_* = 410 mL) for 4 MHz ultrasonic transducer using the fixed-fractional threshold method (blue), AIC method (red) and subjective method of searching for the first zero-crossing (green), in the measurement window (**a**) and zoomed window (**b**).

**Figure 11:**
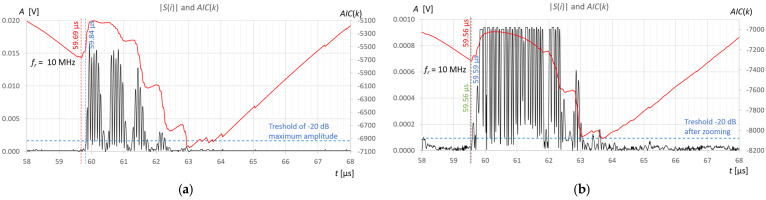
Example results of detecting the start of a pulse reflected from the surface of water in a horizontally placed tank (approximately 45 mm from the bottom, *V_REF_* = 410 mL) for 10 MHz ultrasonic transducer using the fixed-fractional threshold method (blue), AIC method (red) and subjective method of searching for the first zero-crossing (green), in the measurement window (**a**) and zoomed window (**b**).

Measurements and calculations for six fill levels in a horizontally placed tank (Figure 2) were performed using the prototype ultrasonic flaw detector described in Section 2.1, configured with three different ultrasonic sensors (Figure 3). Ultrasonic pulses reflected from the surface of the liquid in the tank were recorded each time in three different measurement time windows: long—covering the transmitted pulse and the pulse reflected from the surface of the liquid (view window), measuring—covering only the reflected pulse, zoomed—covering the reflected pulse in amplitude magnification. Figure 6, Figure 7 and Figure 8 show an example recording of such pulses from water level measurements in the tank (approximately 45 mm from the bottom, which in this case corresponds to a *V_REF_* = 410 mL reference volume) for the three ultrasonic sensors. In the measurement window and in the zoomed window, automatic measurements of the transit time were made using the fixed-fractional threshold method with the amplitude threshold set at 10% (−20 dB) of the maximum amplitude of the pulse reflected from the liquid surface in a given window (Section 2.4) and the AIC method (Section 2.5). Furthermore, in the zoomed window, subjective visual recognition of the beginning of the pulse was performed, ignoring any possible interference, and the first zero-crossing [26] for this pulse was searched for. Figure 9, Figure 10 and Figure 11 show example results of pulse start detection in water for the three ultrasonic sensors. This pulse is also shown in Figure 6b,c, Figure 7b,c and Figure 8b,c.

The ultrasonic pulse return times obtained in this way, reflected on the liquid surface, were converted into the volume of liquid in the tank using the algorithm described in Section 2.3 and compared with the reference volume values *V_REF_* in Table 2 for water and in Table 3 for extraction gasoline.

**Figure 12:**
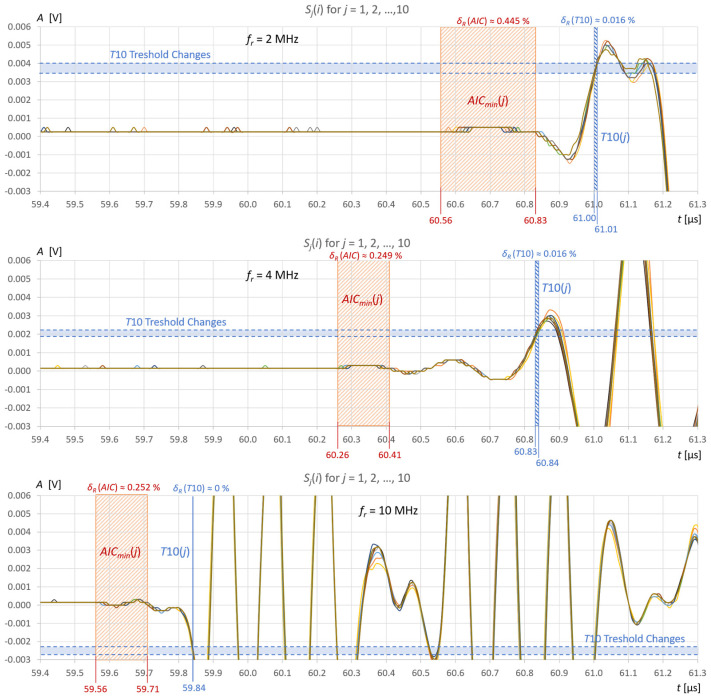
10 recordings of ultrasonic pulses reflected from the surface of 410 mL of water in a tank for 3 ultrasonic sensors with resonance frequencies *f_r_* = 2, 4, and 10 MHz; figures show the spreads of the measured values of the pulse start time using the AIC and T10 methods and the resulting relative percentage errors, as well as the spreads of the detection threshold.

**Figure 13:**
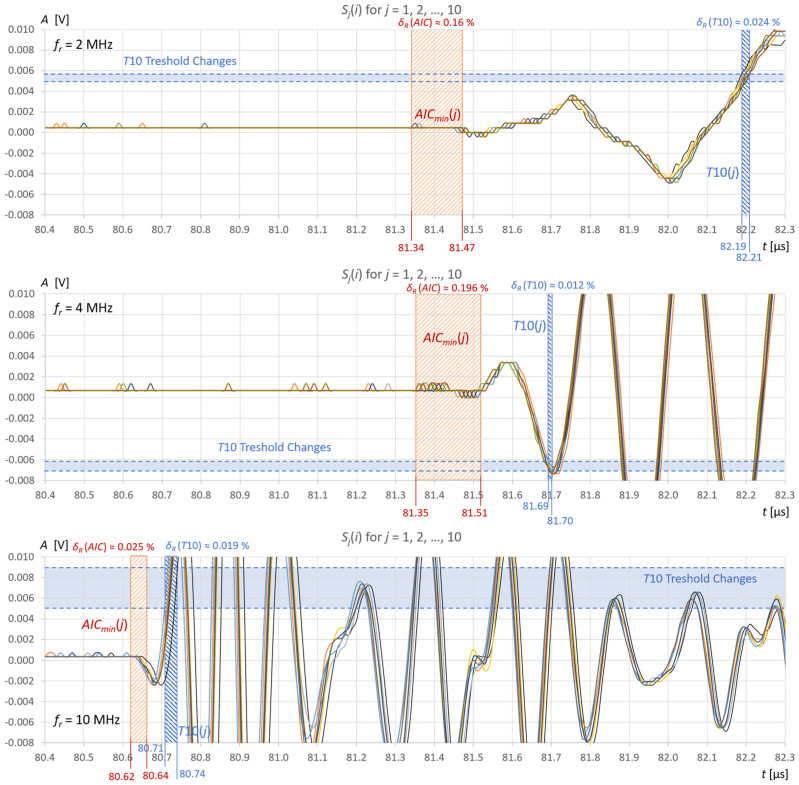
10 recordings of ultrasonic pulses reflected from the surface of 415 mL of extraction gasoline in a tank for 3 ultrasonic sensors with resonance frequencies *f_r_* = 2, 4, and 10 MHz; figures show the spreads of the measured values of the pulse start times using the AIC and T10 methods and the resulting relative percentage errors, as well as the spreads of the detection threshold.

The stability of liquid level detection in the tank under steady measurement conditions was also verified using the AIC method and the fixed-fractional threshold method with a 10% amplitude threshold (T10). Figure 12 and Figure 13 show common graphs of *j* = 10 recordings of ultrasonic pulses reflected from the surface of the water (Figure 12) and the extraction gasoline in the tank (Figure 13) for three ultrasonic sensors.

**Figure 14:**
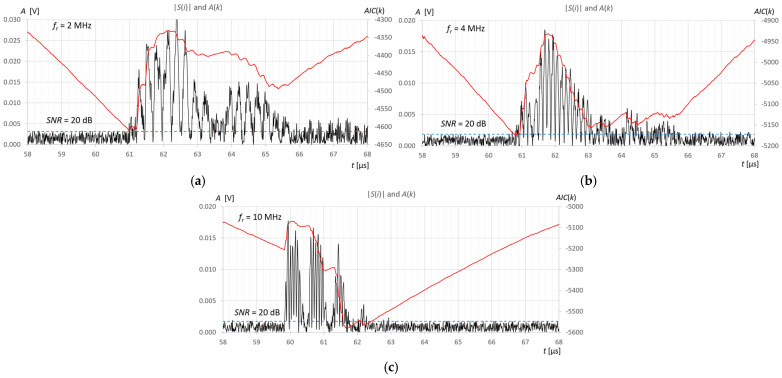
Example results of detecting the start of a pulse reflected from the surface of water in a horizontally placed tank (approximately 45 mm from the bottom, *V_REF_* = 410 mL) with added white noise (SNR = 20 dB), for (**a**) 2 MHz, (**b**) 4 MHz, (**c**) 10 MHz ultrasonic transducer using the T10 method (blue) and the AIC method (red) in the measurement window.

In order to examine the performance of the AIC and TC10 methods in a noisy signal, artificially generated white noise with an amplitude allowing an SNR range of 5 dB to 60 dB with a step of 5 dB was added to the recorded measurement signals. Figure 14 shows an example of pulses reflected from the surface of 415 mL of water in a tank for three ultrasonic sensors and an SNR level value of 20 dB.

## 4. Results

By analyzing the results of the measurements and calculations obtained, it can be concluded that the *AIC*(*k*) function allows a clear minimum to be obtained at the actual start of the ultrasonic pulse in an automatic and universal manner, regardless of the frequency of the measuring sensor. As can be seen in Figure 9b and Figure 11b in the zoomed window, the result of the AIC measurement (red) almost coincides with subjective recognition of the pulse start (green); the differences of approximately 20–30 ns correspond to approximately two to three times the distance between the samples at the applied signal sampling frequency *f_s_* = 100 MHz. The disturbances visible in the zoomed window do not affect the measurement result using the AIC method. In the case of the fixed-fractional threshold method with a 10% threshold set in relation to the upper limit of the zoomed amplitude window (*A* ≈ 0.0004 V), each interference pulse with an amplitude exceeding this threshold significantly distorts the measurement of the start time of the ultrasonic pulse (see Figure 9b and Figure 10b).

However, for automatic measurements, it is easiest to use a standard measurement window that covers the pulse reflected from the liquid surface (Figure 9a, Figure 10a and Figure 11a). The algorithm to set such a time window is simple. It usually involves finding the maximum signal amplitude after the sending pulse (detection of the measured pulse) and setting a window with a fixed number of signal samples before and after the sample with the maximum amplitude. The number of window samples is selected depending on the length of the measured pulse in such a way that signal samples are available for the measurement pulse part, along with a certain number of samples before its start. As can be seen in Figure 9a, Figure 10a and Figure 11a in the standard measurement window, the result of the AIC measurement (red) differs from the subjective recognition of the start of the pulse (green color in Figure 9b, Figure 10b and Figure 11b) by approximately 200 ns, which corresponds to a twenty-fold distance between samples at the applied signal sampling frequency. The measurement error is still small and amounts to approximately +0.33% in relation to the measured time value. When using the threshold method in a standard window with a 10% threshold set in relation to the maximum pulse amplitude (blue color in Figure 9a, Figure 10a and Figure 11a), the algorithm jumps forward by several half-pulse periods, and the T10 measurement result differs from the value of subjective recognition of the pulse start (green in Figure 9b, Figure 10b and Figure 11b) by approximately 300–1600 ns, depending on the frequency of the probe used and the pulse shape. The fixed-fractional threshold method is sensitive to pulse rise time, unlike the AIC method.

In the subjective method of determining the start of the pulse by detecting the first zero-crossing (green color in Figure 9b, Figure 10b and Figure 11b), the difficulty lies in correctly recognizing the first half-cycle of the pulse, which may be hidden in noise and interference.

In Figure 9a and Figure 10a, it is clearly visible, for example, that the measured pulse is elongated due to multiple reflections in the 2-millimeter wall of the tank. However, it does not affect the error in detecting the start of the pulse.

**Figure 15:**
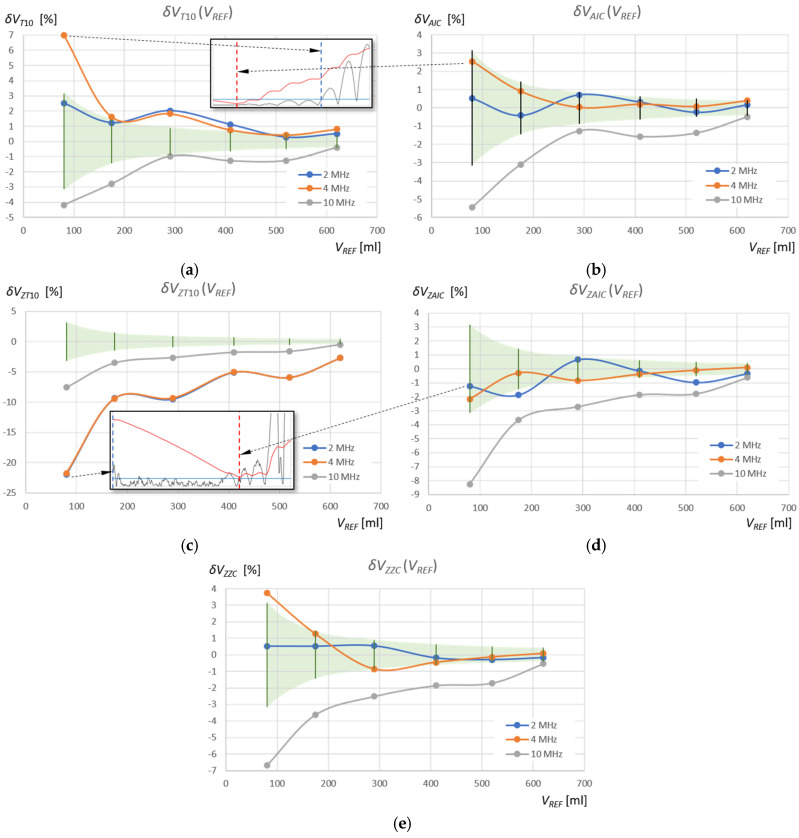
Relative percentage errors in measuring the volume of water in the tank in relation to reference measurements of the tank filling volume *V_REF_* for two detection methods used in the standard measurement window: T10 (**a**) and AIC (**b**), and in the zoomed window ZT10 (**c**), ZAIC (**d**), compared to the subjective ZZC method in the zoomed window (**e**).

**Figure 16:**
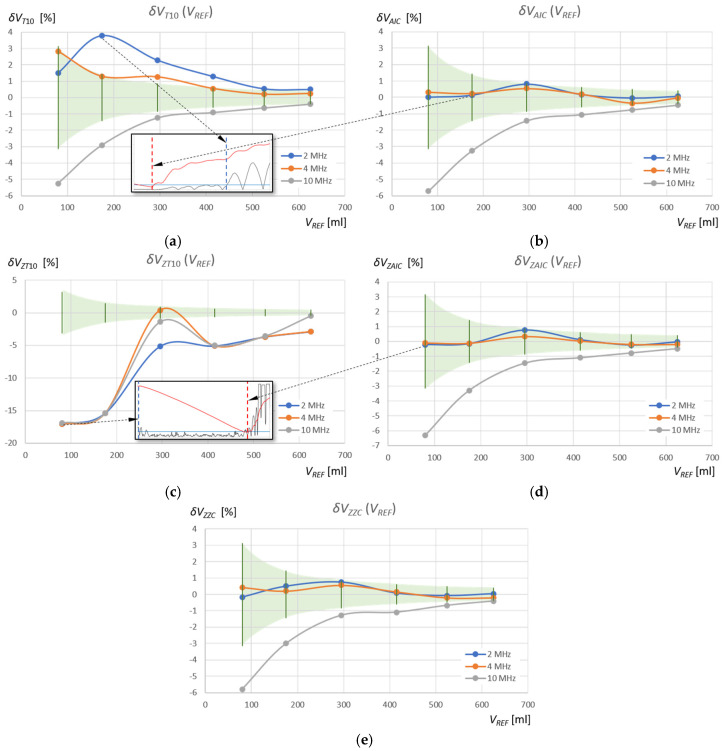
Relative percentage errors in measuring the volume of extraction gasoline in the tank in relation to reference measurements of the tank filling volume *V_REF_* for two detection methods used in the standard measurement window: AIC (**a**) and T10 (**b**), and in the zoomed window ZAIC (**c**), ZT10 (**d**), compared to the subjective ZZC method in the zoomed window (**e**).

From the point of view of measuring the volume of the liquid in a tank, it is important to know how these ultrasonic liquid level measurements translate into the accuracy of the volume measurement. This problem was analyzed by calculating the relative percentage errors in the measurement of the liquid volume in relation to the reference measurements of the filling volume tank (*V_REF_*) using laboratory vessels (Table 2 and Table 3) that allow the measurement of a fixed liquid volume with an accuracy of 5 mL (i.e., Δ*V_REF_* = ±2.5 mL):

(12) $${\delta V}_{WM}=\;\frac{V_{WM}-V_{REF}}{V_{REF}}\cdot 100\%\;,$$

where the index “*WM*” (Window Method) denotes the detection method used in a given measurement window, that is, “T10” and “AIC” in the standard measurement window, and “ZT10,” “ZAIC,” and “ZZC” in the zoomed window (see Table 2 and Table 3). The results of these calculations for the measurements of the volume of water and the volume of the extraction gasoline are shown in Figure 15 and Figure 16, respectively. Additionally, the figures show the relative percentage error of the reference volume measurement for the measured *V_REF_* values (green area), calculated using the following equation:

(13) $${\delta V}_{REF}=\;\frac{∆V_{REF}}{V_{REF}}\cdot 100\%\;.$$

The relative percentage errors in measuring the volume of water in the tank using the AIC method in the standard measurement window (${\delta V}_{AIC}\left(V_{REF}\right)$) fall within the reference volume measurement error range (${\delta V}_{REF}$) for ultrasonic sensors with frequencies of 2 MHz and 4 MHz (Figure 15b), as in the case of the subjective ZZC measurement method in the zoomed window (${\delta V}_{ZZC}\left(V_{REF}\right)$) (Figure 15e), where only 1 measurement for *f_r_* = 4 MHz and *V* = 80 mL is slightly above this range. For the T10 threshold method in the same window ((${\delta V}_{T10}\left(V_{REF}\right)$) and for the same frequencies, there are often significant deviations of several pulse periods (Figure 15a), which is further highlighted by the magnification of the pulse start for the 4 MHz sensor in the measurement of water volume *V_REF_* = 80 mL. As can be seen, these errors are caused by a shift in the detection of the pulse start time as a result of a slow pulse rise time and initial pulse amplitudes below the detection threshold. Therefore, the water volume values measured using the T10 method for frequencies of 2 MHz and 4 MHz are overestimated. The AIC method detects the start of the pulse much more accurately by detecting its very small rise amplitude. In the zoomed measurement window, the errors ${\delta V}_{ZAIC}\left(V_{REF}\right)$ are within the reference volume measurement error range ${\delta V}_{REF}$ for most measurement points and frequencies of 2 MHz and 4 MHz (Figure 15d). However, in this case, the amplitude of the pulse start is at the level of quantization noise, which affects minor detection errors (see the first and the second rows in Figure 12).

In turn, the threshold method in the zoomed measurement window ZT10 for frequencies of 2 MHz and 4 MHz shows significant deviations from the error field ${\delta V}_{REF}$ (Figure 15c), which is further illustrated by the enlargement of the pulse start for the 2 MHz sensor and the water volume measurement *V_REF_* = 80 mL. In this case, disturbances appearing at the beginning of the zoomed measurement window are detected above the threshold. Therefore, the values of the measured water volume using the ZT10 method for frequencies of 2 MHz and 4 MHz are significantly underestimated.

The relative percentage errors in measuring the volume of extraction gasoline in a tank using the AIC method in a standard measurement window (${\delta V}_{AIC}\left(V_{REF}\right)$) oscillate around zero reference volume measurement error (${\delta V}_{REF}$) for ultrasonic sensors with frequencies of 2 MHz and 4 MHz (Figure 16b), as in the case of the subjective ZZC measurement method ((${\delta V}_{ZZC}\left(V_{REF}\right)$) in the zoomed window (Figure 16e). For the T10 threshold method in the same window ((${\delta V}_{T10}\left(V_{REF}\right)$) and for the same frequencies, there are often significant deviations of several pulse periods (Figure 16a), which as in the case of water, is additionally shown in the form of an enlargement of the pulse start for a sensor with a frequency of 2 MHz in the measurement of gasoline volume *V_REF_* = 175 mL. These errors are caused by a shift in the detection of the pulse start time as a result of the slow pulse rise time and the amplitudes of the initial pulse periods lying below the detection threshold, as in the case of water. Therefore, the values of the measured volume of extraction gasoline using the T10 method for frequencies of 2 MHz and 4 MHz are overestimated.

In the case of extraction gasoline, the AIC method very accurately detects the start of the pulse by detection of its very small rise amplitude. In the zoomed window, the errors ${\delta V}_{ZAIC}\left(V_{REF}\right)$ for frequencies of 2 MHz and 4 MHz are equally small and almost the same as the errors ${\delta V}_{AIC}\left(V_{REF}\right)$ in the standard measurement window (compare Figure 16b,d), due to the short rise time of pulses in extraction gasoline. In turn, the threshold method in the zoomed window ZT10 for frequencies of 2 MHz and 4 MHz shows significant deviations from the error field ${\delta V}_{REF}$ (Figure 16c), similar to water, which is additionally highlighted in the form of an enlargement of the pulse start for the 4 MHz sensor in the measurement of the extraction gasoline volume *V_REF_* = 80 mL. In this case, too, disturbances appearing at the beginning of the zoomed window are detected above the threshold, and the values of the measured gasoline volume using the ZT10 method for frequencies of 2 MHz and 4 MHz are significantly underestimated.

**Figure 17:**
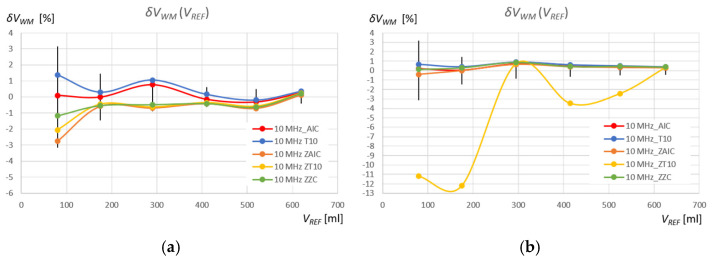
Relative percentage errors in measuring the volume of water (**a**) and the extraction gasoline (**b**) in the tank in relation to the reference measurements of the tank filling volume *V_REF_* for the two detection methods used in the standard measurement window (AIC and T10) and in the zoomed window (ZAIC, ZT10), compared to the subjective ZZC method in the zoomed window for a 10 MHz ultrasonic sensor, all with corrections applied.

It is interesting to note that for a sensor with a frequency of 10 MHz, the percentage volume measurement errors for water and gasoline show a very similar dependence on *V_REF_*, with the exception of the ZT10 method, where the previously discussed errors of detecting random disturbances before the measurement pulse occur (see Figure 15 and Figure 16). The start of the pulse for the 10 MHz frequency is detected very early, which decreases the measured liquid volumes. However, this is the result of a constant systematic error, as can be clearly seen in Figure 12 and Figure 13. The 10 MHz ultrasonic transducer designed for non-destructive material testing is equipped with a fairly thick protective and distancing layer of material with a significantly higher ultrasonic propagation speed than in the tested liquids (Figure 3). This layer accelerates the ultrasonic wave pulse as it passes from the surface of the piezoelectric transducer to the liquid level and back. Multiple reflections in this layer can be seen in the pulse in Figure 11a. Additionally, a thin layer of gel was used to mechanically couple the active surface of the flat probe with the outer surface of the tank wall. If we introduce a correction of +0.7 μs for all pulse return time measurements for a frequency of 10 MHz in water, and +1.0 μs in extraction gasoline, the error values for different methods and measurement windows from Figure 15 and Figure 16 will decrease significantly (see Figure 17), indicating that the best of the tested measurement methods for measuring the volume of liquid in a tank is the AIC method in the standard measurement window for a frequency of 10 MHz (red curve).

**Figure 18:**
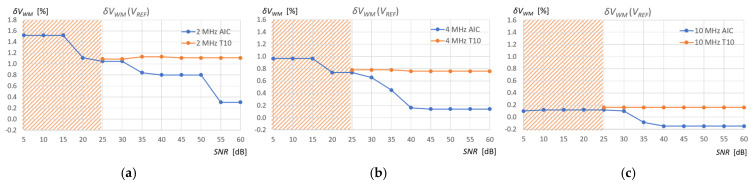
Relative percentage errors in the measurement of the volume of water in the tank depending on the SNR of the measurement signal, for two detection methods (AIC and T10) used in the standard measurement window, and for three ultrasonic sensors: (**a**) 2 MHz, (**b**) 4 MHz, and (**c**) 10 MHz, applied the corrections described above for the 10 MHz sensor.

Figure 18 shows the relative percentage errors in the measurement of the volume of water in the tank relative to the reference measurements of the tank fill volume *V_REF_* (Equation (12)) depending on the SNR of the measurement signal (see Figure 15), for two detection methods (AIC and T10) used in the standard measurement window, and for three ultrasonic sensors (2, 4, 10 MHz), applied the corrections described above for the 10 MHz sensor. The relationships between these errors are similar in the case of the extraction gasoline measurements.

## 5. Discussion

Based on the research conducted in this study, it can be concluded that the use of the AIC method to determine the return time of an ultrasonic pulse reflected from the surface of a liquid in a tank allows its volume to be determined with an accuracy of fractions of a percent. This method is not a threshold method and is based on statistical analysis of signal changes over time. The accuracy obtained in ultrasonic measurements using the AIC method is better than that obtained using the commonly used fixed-fraction amplitude threshold method.

The AIC method is universal and does not show a significant dependence on the applied ultrasonic wave frequency, unlike the threshold method. In the threshold method, the detected pulse start time is always increased due to the need to detect the first half of the absolute amplitude value of the first half-cycle of the pulse exceeding the set threshold above noise and interference. This always results in a liquid volume measurement that is greater than the actual volume. Therefore, the threshold method is sensitive to the shape of the pulse and depends on the value of the set percentage threshold. The longer the pulse rise time, the greater the errors in pulse start detection (delays). In the case of interference with an amplitude exceeding the set detection threshold and occurring before the measured pulse, the threshold method shows variable systematic errors of up to several percent, which in turn decrease the liquid volume by several percent.

Multiple reflections of the ultrasonic pulse in the tank wall (Figure 9a and Figure 10a) or in the protective layer of the sensor (Figure 11a) prolong the recorded receiving pulse and introduce signal envelope modulation. This can cause the global minimum of the *AIC*(*k*) function to shift significantly towards longer TOF, determining the start of the next reflection (Figure 11a). To avoid such errors, the measurement window should be shortened to one pulse period after the maximum detected signal.

In the case of measurements of the volume of extraction gasoline in the tank, more accurate results were obtained than in the case of measurements of the volume of water (see Figure 15 and Figure 16). This is due to the propagation speed of the ultrasonic wave in the extraction gasoline, which is approximately 0.75 times lower than the speed of the ultrasonic wave in water. Measurement errors of larger echo return times in gasoline will therefore be smaller in relation to the shift in the detection of the pulse start by a fixed time (AIC) or a fixed part of its half-periods (TF10).

The repeatability of water level measurements in the tank using the AIC method is 0.202 mm for the 2 MHz sensor and 0.112 mm for the 4 MHz and 10 MHz sensors (Figure 12). Similar measurement accuracy values can be expected, but they cannot be precisely determined because in this study no reference method for measuring liquid levels was used. For the threshold method, the repeatability of the measurements is much better, but the variable systematic error associated with the amplitude threshold ranges is approximately +0.24 μs to +0.53 μs, which translates into a water level measurement error of approximately +0.15 mm for the 10 MHz sensor, +0.38 mm for a 4 MHz sensor, and +0.55 mm for a 2 MHz sensor (Figure 12). The repeatability of the extraction gasoline level measurements in the tank using the AIC method is 0.730 mm for the 2 MHz sensor, 0.090 mm for the 4 MHz sensor, and 0.011 mm for the 10 MHz sensor (Figure 13). For the threshold method, the repeatability of the measurements in the extraction gasoline is significantly better than AIC, but as with the water level measurements, there is a systematic error associated with the amplitude threshold, which is approximately 0.10–0.80 μs, which translates into a level measurement error of approximately +0.05 mm for a 10 MHz sensor +0.15 mm for a 4 MHz sensor, and +0.45 mm for a 2 MHz sensor (Figure 13). The instability of liquid level detection in the tank is mainly due to the influence of liquid surface vibrations (Figure 12 and Figure 13). It can be minimized by averaging several to several dozen recorded signals before measuring the level. This will allow for an additional improvement in measurement accuracy.

Analysis of the performance of the AIC method at different levels of SNR shows greater accuracy and noise immunity compared to the T10 method for the three ultrasonic sensors (Figure 18). Higher frequencies improve the accuracy of both methods, but T10 always overestimates the measured pulse start time values due to the amplitude threshold. The overestimated values measured by the T10 method are independent of the SNR level up to a value slightly above the set amplitude threshold level (in this case, approximately 25 dB), that is, for a given frequency, there is a constant measurement error (Figure 18). Values measured using the T10 method above SNR ≥ 25 dB are random time values that range from the beginning of the measurement window to the beginning of the pulse (see Figure 14), regardless of frequency. This is the result of time measurements for noise samples exceeding the amplitude threshold (orange hatched area in Figure 18). The AIC method performs correct measurements even for very low SNR = 5 dB levels, but the error increases as the SNR decreases. The AIC method shows a weak dependence on the SNR level in the narrow range of 55 to 60 dB for a frequency of 2 MHz, in the range of 40 to 60 dB for 4 MHz, and in the wide range of approximately 5 to 60 dB for 10 MHz. It should be noted that when measuring liquid levels in tanks, the SNR is usually high (more than 40 dB) due to low ultrasonic attenuation, the large surface area of the ultrasonic transducers, and the ultrasonic wave reflection coefficient at the liquid/gas interface being close to 100%.

The computational complexity of the AIC algorithm increases with the length of the signal window to be analyzed. Therefore, a relatively short measurement window should first be defined. This can be done automatically by simple amplitude detection, i.e., by searching for the first absolute maximum of the signal appearing after the transmitted pulse. The measurement window can then be set in terms of the number of samples before and after this maximum, which will ensure that the window covers the samples before and after the start of the measurement pulse. In the case of ultrasonic level measurements of quasi-homogeneous liquids, no echoes or multi-echoes may appear between the transmitted pulse and the pulse reflected from the liquid surface, provided that the repetition frequency of the generated pulses is sufficiently low. The number of mathematical operations required in the AIC algorithm (Equation (7)) for a signal length (measurement window) equal to *N* is: 2 × *N* for logarithm, 4 × *N* for division, *N* × *N* + 2 × *N* for multiplication, 3 × *N* × *N* for summation/subtraction. Logarithmic calculations can be accelerated by tabulating logarithm values, if the measurement system has a suitable memory chip. A simple analysis was performed to assess the applicability of the AIC algorithm to measure liquid levels in real-time embedded systems. Calculating the value of the *AIC*(*k*) function for *N* = 1000 signal samples requires approximately 5 million basic operations. A small processor in the form of a 16-bit microcontroller performs 10 MIPS (Million Instructions Per Second), which means calculating the *AIC*(*k*) function twice per second. Smaller ARM (Advanced RISC Machine) processors used in mobile devices and embedded systems have a computing power of around 0.5 GFLOPS (Giga Floating-Point Operations Per Second), which means calculating the *AIC*(*k*) function 100 times per second. The use of Raspberry Pi 5 SBCs (Small Single-Board Computers) with a computing power of 5 GFLOPS would enable the calculation of the *AIC*(*k*) function 1000 times per second.

## 6. Conclusions

The AIC method is an excellent method for TOF detection in ultrasonic volume measurements in cylindrical tanks. Most false detections occur only between the start of the ultrasonic signal and the first positive zero crossing (Figure 9, Figure 10, Figure 11, Figure 12 and Figure 13). The AIC method provides more accurate results than the commonly used fixed-fractional amplitude threshold method. The development of a more accurate reference method for the direct measurement of liquid volume in a tank could demonstrate even a lower susceptibility of the AIC method to measurement errors in liquid volume using the AIC method. Note that the measurement results are also affected by the accuracy of the ultrasonic wave propagation speed in the liquid used to convert time measurements into distance, which was not considered here.

No influence of the pulse frequency, pulse shape, and above all its rise time was found on the results of pulse start detection using the AIC method.

The AIC method is not sensitive to interference and is capable of detecting the start of a pulse even in noise (Figure 9, Figure 10, Figure 11, Figure 12, Figure 13, Figure 14 and Figure 18). It should be noted that when measuring liquid levels in tanks, the SNR is usually high (more than 40 dB) due to low ultrasonic attenuation, the large surface area of the ultrasonic transducers, and the ultrasonic wave reflection coefficient at the liquid/gas interface being close to 100%.

The most accurate liquid volume measurements using the AIC method were obtained for the ultrasonic sensor with the highest frequency (*f_r_* = 10 MHz); however, due to the additional protective layer on the surface of the transducer, it was necessary to calibrate the measurements by adding a constant time correction value (delay) for the passage of ultrasound through this layer. Such calibration is necessary for every ultrasonic sensor, also due to its design. In these studies, no correlation was found between the results of liquid volume measurements and the diameter of the ultrasonic sensor, but the studies were not strictly conducted in this direction. It should be remembered that the energy and amplitude of the ultrasonic pulse generated in the liquid are higher the larger the active (vibrating) surface area of the piezoelectric transducer. For small diameters and large tanks, the reflected pulse may have a low amplitude, which will not allow an accurate detection of its onset hidden in the noise. In addition, the small diameter and low frequency of the sensor increase the beam divergence angle, and the ultrasonic energy reflected from the liquid surface and returned to the sensor is scattered beyond the sensor surface. Therefore, the selection of an ultrasonic sensor should be carefully carried out, depending on the measurement conditions. Recall that attenuation in a liquid increases with frequency.

The AIC method requires a priori information about the time interval containing the measurement pulse reflected from the liquid surface. Since the SNR level is high in such an application, it is sufficient to search for the maximum signal after the transmission pulse and determine the measurement window on this basis. Since the AIC algorithm selects the arrival time based on the global minimum value for the data window, it should be used in small time intervals that contain a large number of signal samples before the start of the pulse reflected from the liquid surface and a small number of samples after its peak.

The computational complexity of the AIC algorithm has been assessed to be low and it is suitable for real-time liquid level measurement. Information about TOF is carried here by the ultrasonic signal in the current measurement rather than signals in two consecutive measurements. Therefore, the performance of the AIC method is not limited by the waveform similarity between two measurements. The advantages of the AIC method are the high accuracy, versatility, elimination of complex calculations, the ease of implementation, and the suitability for embedded systems.

## Figures and Tables

**Table 1:** The coefficients of Formula (1).

*i*	*k_i_* [m/s]
0	1402.385
1	5.038813
2	−5.799136 × 10^−2^
3	3.287156 × 10^−4^
4	−1.398845 × 10^−6^
5	2.787860 × 10^−9^

**Table 2:** The volume of water in the tank determined on the basis of ultrasonic measurements compared to a given reference volume *V_REF_*, where *f_r_*—resonance frequency of the ultrasonic transducer used in the measurements, *V_T_*_10_—volume determined by the fixed-fractional method with a threshold of −20 dB in the measurement window, *V_ZT_*_10_—volume determined by the fixed-fractional method with a threshold of −20 dB in the zoomed window, *V_AIC_*—volume determined by the AIC method in the measurement window, *V_ZAIC_*—volume determined by the AIC method in the zoomed window, *V_ZZC_*—volume determined by the subjective method of searching for the first zero-crossing in the zoomed window.

*V_REF_* [mL]	*f_r_* [MHz]	*V_T_*_10_ [mL]	*V_AIC_* [mL]	*V_ZT_*_10_ [mL]	*V_ZAIC_* [mL]	*V_ZZC_* * [mL]
80	2	82.016	80.415	62.450	79.014	80.415
	4	85.592	82.036	62.574	78.268	83.001
	10	76.652	75.645	73.953	73.391	74.641
175	2	177.145	174.275	158.559	171.724	175.903
	4	177.823	176.580	158.539	174.486	177.201
	10	170.118	169.579	168.810	168.579	168.656
290	2	295.854	292.065	262.442	291.897	291.560
	4	295.299	290.080	262.972	287.558	287.474
	10	287.171	286.331	282.302	282.134	282.721
410	2	414.547	411.257	389.172	409.399	411.088
	4	413.113	410.581	389.341	408.386	408.132
	10	404.752	403.484	402.638	402.385	402.385
520	2	521.421	518.733	489.132	514.927	518.496
	4	522.237	520.421	489.082	519.473	519.315
	10	513.444	512.808	511.454	510.736	511.135
620	2	623.132	621.015	603.210	617.882	618.952
	4	625.039	622.472	603.210	620.617	620.484
	10	617.547	616.944	616.809	616.137	616.675

* Subjectively determined.

**Table 3:** The volume of extraction gasoline in the tank determined on the basis of ultrasonic measurements compared to a given reference volume *V_REF_* (designations as in Table 2).

*V_REF_* [mL]	*f_r_* [MHz]	*V_T_*_10_ [mL]	*V_AIC_* [mL]	*V_ZT_*_10_ [mL]	*V_ZAIC_* [mL]	*V_ZZC_* * [mL]
80	2	81.204	80.009	66.332	79.818	79.865
	4	82.260	80.247	66.332	79.913	80.343
	10	75.793	75.417	66.468	74.948	75.370
175	2	181.635	175.234	148.068	174.712	175.872
	4	177.265	175.408	148.068	174.77	175.350
	10	169.917	169.284	148.068	169.226	169.744
295	2	301.754	297.400	279.793	297.274	297.211
	4	298.725	296.580	296.139	295.95	296.643
	10	291.351	290.785	290.911	290.659	291.225
415	2	420.370	415.706	393.883	415.4536	415.327
	4	417.282	415.769	393.883	415.075	415.643
	10	411.225	410.593	394.200	410.403	410.467
525	2	527.83	524.774	505.575	523.654	524.597
	4	526.068	523.065	505.575	523.890	523.890
	10	521.707	520.998	506.294	520.880	521.471
625	2	628.100	625.365	606.843	624.776	625.267
	4	626.540	624.678	606.843	623.694	623.694
	10	622.509	622.014	622.113	621.915	622.410

* Subjectively determined.

## Data Availability

Data supporting reported results can be obtained from the authors by special request. The data is not stored in the cloud or publicly available.

## Abbreviations

AIC	Akaike Information Criterion
BIC	Bayesian Schwartz Criterion
TOF	Time-Of-Flight
SNR	Signal-To-Noise Ratio
ZC	Zero-Crossing
MIPS	Million Instructions Per Second
ARM	Advanced RISC Machine
GFLOPS	Giga Floating-Point Operations Per Second
SBCs	Small Single-Board Computers
UTT	Ultrasound Transmission Tomography
